# Effectiveness of call system implementation for postpartum hemorrhage in a tertiary emergency medical center: a retrospective cohort study

**DOI:** 10.1186/s12884-023-06095-2

**Published:** 2023-11-11

**Authors:** Sayo Umeda, Takeru Abe, Soichiro Obata, Shigeru Aoki, Ichiro Takeuchi

**Affiliations:** 1 Perinatal Center for Maternity and Neonates, Yokohama City University Medical Center, Yokohama, Japan; 2 Advanced Critical Care and Emergency Center, Yokohama City University Medical Center, Yokohama, Japan; 3 Department of Emergency Medicine, Graduate School of Medicine, Yokohama City University, Yokohama, Japan

**Keywords:** Postpartum hemorrhage, Mortality, Clinical protocol, Patient care team

## Abstract

**Background:**

Postpartum hemorrhage is the leading cause of maternal death and severe maternal morbidity worldwide. Previous studies have reported the importance of multidisciplinary treatment approaches for postpartum hemorrhage; however, only a few studies have shown a clear improvement in maternal outcomes. Therefore, this study aimed to investigate the efficacy of a call system for postpartum hemorrhage in a tertiary emergency facility for rapid multidisciplinary treatment and its effect on maternal outcomes.

**Methods:**

This single-center retrospective cohort study included patients transferred to our hospital due to postpartum hemorrhage between April 1, 2013, and March 31, 2019. The primary outcome was mortality, and the secondary outcomes were morbidity (duration of hospital stay, duration of intensive care unit stay, admission to the intensive care unit, respirator use, duration of ventilator support, acute kidney injury, transfusion-associated circulatory overload/transfusion-related acute lung injury, hysterectomy, composite adverse events, blood transfusion initiation time, blood transfusion volume, and treatment for postpartum hemorrhage). An in-hospital call system implementation commenced on April 1, 2016. The study outcomes were compared 3 years before and after implementing the call system.

**Results:**

The blood transfusion initiation time and duration of hospital stay were significantly shortened after implementing the call system for postpartum hemorrhage. No maternal deaths were observed after implementing the system.

**Conclusions:**

Implementing call systems specialized for postpartum hemorrhage in tertiary emergency facilities may improve maternal outcomes.

## Background

Postpartum hemorrhage (PPH) occurs in approximately 1–3% of all births, although its incidence varies by region and patient background [1–3]. PPH is responsible for one-quarter of maternal deaths globally [3, 4], with approximately 140,000 deaths annually [5]. Furthermore, it is the leading cause of maternal death and severe maternal morbidity worldwide [6–8]. An increase in PPH incidence has been reported in both low- and high-income countries [9], as well as an increase in its severity [10]. Previous studies have mainly focused on the importance of a multidisciplinary team approach for PPH [11–13].

The causes of PPH vary widely, including uterine atony, surgical trauma or tears, placenta previa or accreta, and coagulation disorders [14, 15]. The Maternal Death Exploratory Committee of the Japanese Society of Obstetrics and Gynecology reported that half of the deaths due to obstetric hemorrhage in Japan were preventable [16]. Furthermore, more than half of deliveries in Japan are managed in private clinics; hence, cooperation and coordination between regional hospitals are necessary to address PPH [16].

In treating PPH, it is necessary to select the appropriate hemostatic method according to the etiology and general condition of patients. Hemorrhagic shock or disseminated intravascular coagulation (DIC) is common in patients with PPH, and facilitating the integration and timely escalation of pharmacologic, radiological, surgical, and transfusion interventions are critical to successfully managing this condition [6]. Multidisciplinary treatment with call systems may accelerate treatment initiation and consequently contribute to patient outcomes. Hence, it is important to create a standardized algorithm for PPH treatment in each institution [17].

Some studies have examined whether implementing a multidisciplinary algorithm for PPH treatment changes important outcomes, such as maternal mortality, hysterectomy due to bleeding, intensive care unit (ICU) admission, and transfusion-associated circulatory overload (TACO) [18–22]. However, there are limited results with high-quality evidence.

**Fig. 1:**
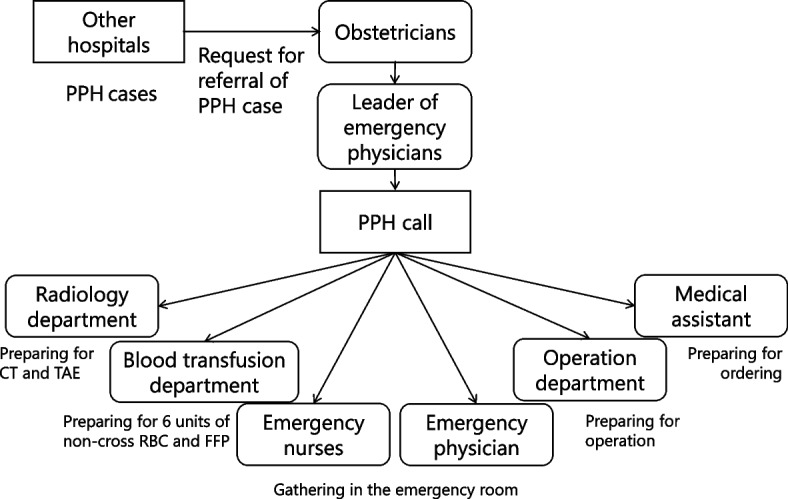
In-hospital call system (PPH call) PPH, postpartum hemorrhage; CT, computed tomography; TAE, transcatheter arterial embolization; RBC, red blood cell; FFP, fresh frozen plasma

Based on this background, from 2016 onward, an in-hospital call system known as “PPH call” was implemented for patients transferred to our hospital due to PPH. This call system aided the rapid initiation of multidisciplinary treatment for transported patients with PPH (Fig. 1). Therefore, this study aimed to investigate the efficacy of a call system for PPH in a tertiary emergency facility for rapid multidisciplinary treatment and maternal outcomes.

## Methods

### Study design and participants

This was a single-center retrospective cohort study. It included all patients transferred to our hospital due to PPH from April 1, 2013, to March 31, 2019. The “PPH call” implementation commenced on April 1, 2016, and the characteristics of the patients transferred before and after the implementation were compared.

### PPH call

The “PPH call” is an in-hospital call system triggered when obstetricians in other hospitals request the transfer of patients with PPH to the obstetrics unit at our hospital via a phone call, and obstetricians in our facility accept the request (Fig. 1). When the PPH call is triggered, all relevant departments are notified simultaneously. Next, the emergency physicians, emergency nurses, and obstetrics staff gather in the emergency room, and the case is registered in the medical records for immediate treatment. The blood transfusion department prepares six units of non-cross red blood cells and six units of fresh frozen plasma (FFP). In addition, the anesthesiologists, operation room staff, and radiology departments are notified that a PPH case has been referred to the hospital. Radiologists are always present during the daytime and are notified simultaneously, like other departments. However, they are called at night if necessary based on the patient's condition and arrive at the hospital within 30 min. Before the patient arrives at the hospital, a multidisciplinary briefing with all teams involved occurs in the emergency room to share information about the patient and the treatment plan. Hence, the call system enables the prompt initiation of multidisciplinary treatment upon patient arrival.

### Study outcomes

The primary outcome was maternal mortality. The secondary outcomes were the duration of hospital stay, duration of ICU stay, admission to the ICU, respirator use, duration of ventilator support, acute kidney injury (AKI), TACO/transfusion-related acute lung injury (TRALI), hysterectomy, composite adverse events, blood transfusion initiation time, blood transfusion volume, and treatment for PPH. Composite adverse events were defined as including death, ICU admission, respirator use, TACO/TRALI, AKI, and hysterectomy.

### Background and laboratory data collection and definitions

The data collected included patient age, delivery method, shock index (defined as heart rate divided by systolic blood pressure), fibrinogen level, presence of DIC, hemoglobin level, platelet count, and lactate level on admission. Fibrinogen levels were measured at a central laboratory without point-of-care testing. Based on the obstetric DIC score [23], DIC was defined as fibrinogen levels ≤ 150 mg/dL on admission.

The obstetric DIC score is determined based on the underlying obstetric disease and clinical and laboratory findings. This score is useful for performing treatment before laboratory findings are known while considering the risk of DIC [23]. Therefore, in our hospital, if DIC is suspected based on clinical findings and the obstetric DIC score, DIC treatment is commenced before the fibrinogen level result is available. Of the laboratory findings used to determine the obstetric DIC score, fibrinogen levels have been reported to be useful for predicting DIC [24]. Additionally, in Japan, it is recommended to administer fibrinogen preparations to patients with fibrinogen levels ≤ 150 mg/dL.

### Statistical analysis

The association between the implementation of the PPH call and each item was examined using the Mann–Whitney U test for continuous variables and Fisher's exact test for categorical variables. In addition, multiple regression analysis with backward selection was used to examine the effect of the PPH call implementation on the duration of hospital stay after adjusting for covariates. IBM-SPSS ver. 25 (IBM Corp., Armonk, NY, USA) was used for all statistical analyses. Two-sided *p*-values < 0.05 were considered statistically significant. Because this was a retrospective study, a power analysis was performed instead of a sample size calculation, which is for prospective studies. From the power analysis, when the partial regression coefficient was 0.5 with 111 samples, a power of 0.999 was obtained.

### Ethical statement

This study was performed in accordance with the Declaration of Helsinki guidelines and approved by the Ethics Committee/institutional review board (IRB) of Yokohama City University Medical Center (approval number: B170300025). The need for informed consent from patients was waived by the Ethics Committee/IRB of Yokohama City University Medical Center because of the study’s retrospective design.

## Results

### Patient background and laboratory data

There were 45 and 66 eligible patients before and after call implementation, respectively. Table 1 shows the patients’ backgrounds and laboratory data upon admission to our hospital. Mean fibrinogen levels were significantly lower after the call implementation than before. Therefore, the presence of DIC (defined as fibrinogen < 150 mg/dL) at admission tended to be higher after call implementation than before, although there was no significant difference. No significant differences in other items were observed between patients admitted before and after call implementation.

**Table 1:** Patient background and laboratory data on hospital admission

	Before ^a^ (*n* = 45)		After ^b^ (*n* = 66)		
	median (interquartile range) or frequency (%)		median (interquartile range) or frequency (%)		*p*-value
Age; years	32	(32.0–38.0)	33	(29.0–36.0)	0.845
Cesarean section	9	(20%)	6	(9%)	0.100
Shock index	0.842	(0.707–1.044)	0.800	(0.667–1.008)	0.455
Fibrinogen; mg/dL	325	(174–397)	217	(157–339)	0.042
Presence of DIC	5	(11%)	14	(21%)	0.205
Hemoglobin; g/dL	7.7	(6.0–9.5)	7.8	(6.6–9.5)	0.281
Platelet; 10^×4^	14.9	(9.6–19.9)	17.9	(12.2–21.4)	0.099
Lactate; mmol/L	3.0	(2.0–4.9)	3.1	(2.0–4.4)	0.877

Before ^a^, before PPH call implementation; After ^b^, after PPH call implementation

DIC, disseminated intravascular coagulation

### Comparison of treatments and outcomes before and after PPH call implementation

Table 2 shows a comparison of maternal outcomes and hospital courses before and after PPH call implementation. While no maternal death was observed after implementing the PPH call, one death was recorded before its implementation. Regarding secondary outcomes, significant reductions in the duration of hospital stay (from 6 to 4 days; *p* = 0.016) and the transfusion initiation time (from 68.5 to 16 min; *p* < 0.001) were observed. The incidence of AKI decreased by two-thirds from 16 to 5% after call system implementation, although this difference was not statistically significant. No significant differences in other morbidity parameters, such as hysterectomy and TACO/TRALI, were observed. Further, no significant differences in the incidence of composite adverse events, including death, ICU admission, respirator use, TACO/TRALI, AKI, and hysterectomy, were observed before and after call implementation. Furthermore, patients were grouped according to the PPH treatments they received as (i) patients that underwent only transcatheter arterial embolization (TAE), (ii) those that required some type of operation, such as a perineal laceration suture or total hysterectomy, and (iii) those that received only conservative treatments with uterotonic drugs, intrauterine balloon, fluid transfusion, or blood transfusion. Then, the outcomes of the groups were compared; however, no significant difference was observed. Additionally, no significant difference in the use of uterotonic drugs such as oxytocin and methylergometrine, or intrauterine balloons, was observed before and after the call implementation. These were sometimes used in combination with other treatments.

**Table 2:** Comparison of maternal outcomes and hospital courses before and after PPH call implementation

	Before ^a^ (*n* = 45)		After ^b^ (*n* = 66)		
	median (interquartile range) or frequency (%)		median (interquartile range) or frequency (%)		*p*-value
Maternal death	1	(2%)	0	(0%)	> 0.99
Duration of hospital stay; days	6	(3.0–10.0)	4	(2.0–7.0)	0.016
Duration of ICU stay; days	0	(0.0–1.5)	0	(0.0–1.3)	0.686
Admission to ICU	14	(31%)	17	(26%)	0.667
Duration of ICU admission; days	2	(1.8–2.0)	2	(2.0–2.8)	0.294
Respirator use	12	(27%)	12	(19%)	0.482
Duration of ventilator support; days	0	(0.0–1.0)	0	(0.0–0.0)	0.367
AKI	7	(16%)	3	(5%)	0.087
TACO/TRALI	0	(0%)	0	(0%)	1.000
Hysterectomy	3	(7%)	5	(8%)	1.000
Composite adverse events	20	(44%)	20	(30%)	0.160
Blood transfusion initiation time; min	68.5	(31.5–144.3)	16.0	(9.0–66.0)	< 0.001
RBC volume; U	6	(2.0–10.0)	6	(1.5–12.0)	0.825
FFP volume; U	4	(0.0–11.0)	4	(0.0–12.0)	0.898
Treatment					0.485
Conservative treatment	15	(33%)	19	(29%)	
TAE alone	9	(20%)	9	(14%)	
Operation	21	(47%)	38	(58%)	
Oxytocin	23	(51%)	39	(59%)	0.441
Methylergometrine	6	(13%)	8	(12%)	1.000
Uterine balloon	5	(11%)	6	(9%)	0.755

Before ^a^, before PPH call implementation; After ^b^, after PPH call implementation

*ICU* intensive care unit, *AKI* acute kidney injury, *TACO* transfusion-associated circulatory overload, *TRALI* transfusion-related acute lung injury, *RBC* red blood cell, *FFP* fresh frozen plasma, *TAE* transcatheter arterial embolization

Since tranexamic acid administration was not recommended for PPH treatment in Japan during the study period, it was not administered to any of the patients in this study. Additionally, compression sutures and uterine artery ligation were not employed in any of the cases because TAE could be performed at any time in our facility, and it was the first-line treatment in patients who were potential candidates for compression sutures or uterine artery ligation. Furthermore, fibrinogen concentrate was not covered by public insurance in Japan during the study period. Therefore, fibrinogen concentrate could not be administered to patients admitted before the PPH call implementation. However, after the call implementation, it was administered in PPH cases with suspected hypofibrinogenemia after obtaining approval from the Ethics Committee/IRB of Yokohama City University Medical Center. Notably, a cryoprecipitate was not administered at our hospital.

### Factors associated with the duration of hospital stay before and after PPH call implementation

The results of the multivariate analysis of confounding factors for the duration of hospital stay are shown in Table 3. Immediate blood transfusion initiation (*p* = 0.001) and PPH call implementation (*p* = 0.049) were significantly associated with a shorter duration of hospital stay. Conversely, FFP volume (*p* < 0.001) was significantly associated with a prolonged hospital stay. The coefficient of determination for the model was 0.50 (adjusted 0.45).

**Table 3:** Multivariate analysis of confounding factors for the duration of hospital stay

	Partial regression coefficient	Standard error	Standardized partial regression coefficient	*p*-value
Age	0.17	0.08	0.20	0.031
Blood transfusion initiation time	0.00	0.00	0.31	0.001
FFP volume	0.18	0.04	0.38	< 0.001
Call implementation	-1.63	0.81	-0.19	0.049
Coefficient of determination	0.50	(0.45)		

*FFP* Fresh frozen plasma

### Comparison of PPH etiology before and after call implementation

**Fig. 2:**
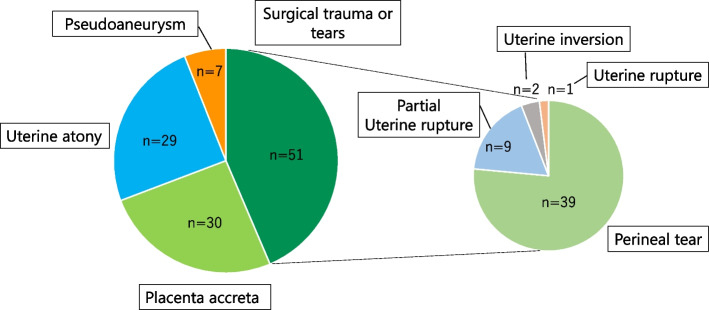
Categorization of the causes of postpartum hemorrhage

The causes of PPH were categorized as surgical trauma or tears, placenta previa or acreta, uterine atony, or pseudoaneurysm (Fig. 2). No PPH cases occurred due to placenta previa and coagulation disorders, and no differences in the causes of PPH were observed before and after call implementation.

### Neonatal outcomes

One preterm birth occurred before call implementation, and two preterm births and one mid-term miscarriage occurred after call implementation.

## Discussion

This study investigated and clarified the efficacy of a call system for PPH in a tertiary emergency facility for rapid multidisciplinary treatment and its effect on maternal outcomes. After implementing the call system, the blood transfusion initiation time and the duration of hospital stay were significantly shortened. One maternal death occurred before the implementation of the call system; however, no maternal deaths were observed after implementation. Therefore, implementing a call system for PPH may improve the outcomes of patients with PPH.

Hasegawa et al., from the Maternal Death Exploratory Committee of the Japanese Society of Obstetrics and Gynecology, reported that about half of the maternal deaths caused by PPH in Japan could be prevented by prompt diagnosis and treatment, including the establishment of a system in addition to maternal transport, intervention, and blood transfusion [20]. The PPH call in this study is designed to notify all relevant departments when the decision is made to transport a patient to our hospital, thus allowing the quick mobilization of personnel and all necessary medical resources (Fig. 1). All team members involved in the patient’s treatment gather for a briefing to share information about the patient and their treatment plan. Blood products are prepared beforehand and promptly administered as needed when the patient arrives at the hospital. Therefore, multidisciplinary treatment can be promptly initiated upon the patient’s arrival at the hospital. In addition, the system may lead to a quicker acceptance of patients for transport, resulting in shorter transportation times. Although the number of cases was small and no significant differences in maternal death were observed before and after implementing this system, no deaths occurred after its implementation. This observation suggests that the call system may have contributed to the reduced mortality rates, and further studies with larger sample sizes may show significant differences.

With the implementation of the call system, the time from arrival at the hospital to the start of blood transfusion (from 68.5 to 16 min) and the duration of the hospital stay (from 6 to 4 days) were significantly shortened (Table 2). No significant differences were observed in the incidence rates of AKI, hysterectomy, and TACO/TRALI, which are considered morbidity parameters. Multivariate analysis of factors associated with shorter hospital stays showed that transfusion initiation time and call implementation contributed to shorter hospital stays, even after accounting for confounding factors (Table 3). A previous study reported that early FFP transfusion for patients with PPH did not improve maternal outcomes compared to those who received no or late FFP transfusion [25]. However, the study may not have included PPH cases with severe coagulopathy. Contrastingly, the present study revealed that early transfusions administered as needed were associated with a shorter hospital stay. In particular, the standardized partial regression coefficient for the reduction in transfusion initiation time was large, suggesting that it significantly influenced the reduction in the duration of hospital stay. Furthermore, the FFP volume was significantly associated with a prolonged duration of hospital stay. This may have been because patients who required more FFP volume had more severe PPH, thus requiring longer hospitalization. However, this association is not important in this study because no difference in FFP volume before and after call implementation was observed. Therefore, it can be deduced that the impact of multidisciplinary treatment through call implementation, including prompt blood transfusion initiation, on shortening the duration of hospital stay is greater than that of the FFP volume on prolonging the duration of hospital stay. Implementing this call system, which enabled simultaneous dissemination of information to all relevant departments and the prompt provision of necessary medical resources, may have led to a marked reduction in the time required for blood transfusion initiation. In turn, this may have prevented maternal death and reduced the duration of hospital stay.

Protocol development is essential to ensure quality and safe medical care; this also applies to perinatal management in reducing adverse obstetrical outcomes [26]. Protocols with standardized interventions improve emergency care. Hence, similar to cardiac arrest management [27], standardized management plans should be developed for PPH. Furthermore, teamwork training has been reported to improve knowledge, practical skills, communication, and team performance [12]. The call system in the present study may improve patient outcomes, as the obstetrics and emergency departments share the same treatment plan and communicate smoothly with the surgery, radiology, and blood transfusion departments.

This study had some limitations. First, this was a single-center retrospective cohort study; hence, adapting the call system in this study to other facilities may be difficult. However, developing a protocol for PPH management in each region is important. Therefore, modifying the system for adaptation in each facility based on considerations about which treatments can be performed and assessment of the ease of transportation to tertiary facilities may improve maternal outcomes. Second, the sample size of this study was small; thus, the analyses might have been underpowered to detect significant differences in the outcomes. Although the before-after comparison of this novel treatment protocol and our findings show promise, further studies are needed to identify causal relationships between the study variables and the outcomes. Third, this was a comparative study of cases before and after the implementation of the call system. Thus, the impact of improved medical care due to increased clinical experience and changing treatment strategies, such as fibrinogen concentrate administration, cannot be excluded. Previous studies have suggested that fibrinogen concentrate substitution therapy for PPH increases fibrinogen levels and reduces bleeding [28–30]. However, since fibrinogen concentrate could only be administered after call implementation at our hospital, its effects could not be compared. Finally, pursuing the validity of this study’s findings in a multicenter study may be necessary.

Protocols for preventing and treating PPH [3, 21] and a multidisciplinary team approach have been reported to be important for successful PPH management [11–13]. However, limited data exist on the improvement in maternal outcomes. This study found that implementing a call system for PPH in a tertiary emergency facility contributed to shorter transfusion initiation times and duration of hospital stays. Therefore, the results of this study may provide recommendations that can be applied to other facilities.

## Conclusions

Implementing a call system specialized for PPH in a tertiary emergency facility may improve maternal outcomes despite an increased incidence of hypofibrinogenemia on hospital admission.

## Data Availability

The datasets used and/or analyzed during the current study are available from the corresponding author upon reasonable request.

## Abbreviations

PPH: Postpartum hemorrhage
DIC: Disseminated intravascular coagulation
ICU: Intensive care unit
TACO: Transfusion-associated circulatory overload
TRALI: Transfusion-related acute lung injury
AKI: Acute kidney injury
TAE: Transcatheter arterial embolization
RBC: Red blood cell
FFP: Fresh frozen plasma
CT: Computed tomography

## References

[1] Sheldon WR, Blum J, Vogel JP, Souza JP, Gülmezoglu AM, Winikoff B (2014). Postpartum haemorrhage management, risks, and maternal outcomes: findings from the World Health Organization Multicountry Survey on Maternal and Newborn Health. BJOG.

[2] Reale SC, Easter SR, Xu X, Bateman BT, Farber MK (2020). Trends in Postpartum Hemorrhage in the United States From 2010 to 2014. Anesth Analg.

[3] World Health Organization. WHO Recommendations for the Prevention and Treatment of postpartum haemorrhage [WHO website]; 2012. https://apps.who.int/iris/bitstream/handle/10665/75411/9789241548502_eng.pdf?sequence=1. Accessed 31 Aug 2021. 23586122

[4] Say L, Chou D, Gemmill A, Tunçalp Ö, Moller AB, Daniels J (2014). Global causes of maternal death: a WHO systematic analysis. Lancet Glob Health.

[5] Dahlke JD, Mendez-Figueroa H, Maggio L, Hauspurg AK, Sperling JD, Chauhan SP (2015). Prevention and management of postpartum hemorrhage: a comparison of 4 national guidelines. Am J Obstet Gynecol.

[6] Baird EJ (2017). Identification and Management of Obstetric Hemorrhage. Anesthesiol Clin.

[7] Maswime S, Buchmann E (2017). A systematic review of maternal near miss and mortality due to postpartum hemorrhage. Int J Gynaecol Obstet.

[8] Rath WH (2011). Postpartum hemorrhage–update on problems of definitions and diagnosis. Acta Obstet Gynecol Scand.

[9] van Stralen G, von Schmidt Auf Altenstadt JF, Bloemenkamp KW, van Roosmalen J, Hukkelhoven CW. Increasing incidence of postpartum hemorrhage: the Dutch piece of the puzzle. Acta Obstet Gynecol Scand. 2016;95:1104–10. 10.1111/aogs.12950 27460955

[10] Kramer MS, Berg C, Abenhaim H, Dahhou M, Rouleau J, Mehrabadi A (2013). Incidence, risk factors, and temporal trends in severe postpartum hemorrhage. Am J Obstet Gynecol.

[11] de Vries PLM, Deneux-Tharaux C, Baud D, Chen KK, Donati S, Goffinet F, et al. Postpartum haemorrhage in high-resource settings: Variations in clinical management and future research directions based on a comparative study of national guidelines. BJOG. 10.1111/1471-0528.17551 10.1111/1471-0528.17551 37259184

[12] The Royal Australian and New Zealand College of Obstetricians and Gynaecologists. Management of Postpartum Haemorrhage (PPH) [RANZCOG website]. 2016. https://ranzcog.edu.au/RANZCOG_SITE/media/RANZCOG-MEDIA/Women%27s%20Health/Statement%20and%20guidelines/Clinical-Obstetrics/Management-of-Postpartum-Haemorrhage-(C-Obs-43)-Review-July-2017.pdf?ext=.pdf. Accessed 31 Aug 2021.

[13] Royal College of Obstetricians and Gynaecologists. Prevention and Management of Postpartum Haemorrhage: Green-top Guideline No. 52. BJOG. 2017;124:e106-e149. 10.1111/1471-0528.14178 27981719

[14] Kolin DA, Shakur-Still H, Bello A, Chaudhri R, Bates I, Roberts I (2020). Risk factors for blood transfusion in traumatic and postpartum hemorrhage patients: Analysis of the CRASH-2 and WOMAN trials. PLoS ONE.

[15] Laganà AS, Sofo V, Salmeri FM, Chiofalo B, Ciancimino L, Triolo O (2015). Post-partum management in a patient affected by thrombotic thrombocytopenic purpura: case report and review of literature. Clin Exp Obstet Gynecol.

[16] Hasegawa J, Sekizawa A, Tanaka H, Katsuragi S, Osato K, Murakoshi T (2016). Current status of pregnancy-related maternal mortality in Japan: a report from the Maternal Death Exploratory Committee in Japan. BMJ Open.

[17] Main EK, Goffman D, Scavone BM, Low LK, Bingham D, Fontaine PL (2015). National Partnership for Maternal Safety: consensus bundle on obstetric hemorrhage. Anesth Analg.

[18] Ueda A, Nakakita B, Chigusa Y, Mogami H, Ohtera S, Kato G (2022). Impact of efforts to prevent maternal deaths due to obstetric hemorrhage on trends in epidemiology and management of severe postpartum hemorrhage in Japan: a nationwide retrospective study. BMC Pregnancy Childbirth.

[19] McNamara H, Kenyon C, Smith R, Mallaiah S, Barclay P (2019). Four years' experience of a ROTEM® -guided algorithm for treatment of coagulopathy in obstetric haemorrhage. Anaesthesia.

[20] Bell SF, Collis RE, Pallmann P, Bailey C, James K, John M (2021). Reduction in massive postpartum haemorrhage and red blood cell transfusion during a national quality improvement project, Obstetric Bleeding Strategy for Wales, OBS Cymru: an observational study. BMC Pregnancy Childbirth.

[21] Khanna P, Sinha C, Singh AK, Kumar A, Sarkar S (2023). The role of point of care thromboelastography (TEG) and thromboelastometry (ROTEM) in management of Primary postpartum haemorrhage: A meta-analysis and systematic review. Saudi J Anaesth.

[22] Skupski DW, Brady D, Lowenwirt IP, Sample J, Lin SN, Lohana R (2017). Improvement in Outcomes of Major Obstetric Hemorrhage Through Systematic Change. Obstet Gynecol.

[23] Kobayashi T (2014). Obstetrical disseminated intravascular coagulation score. J Obstet Gynaecol Res.

[24] Morikawa M, Matsunaga S, Makino S, Takeda Y, Hyoudo H, Nii M (2021). Effect of hypofibrinogenemia on obstetrical disseminated intravascular coagulation in Japan in 2018: a multicenter retrospective cohort study. Int J Hematol.

[25] Henriquez DDCA, Caram-Deelder C, le Cessie S, Zwart JJ, van Roosmalen JJM, Eikenboom JCJ (2019). Association of Timing of Plasma Transfusion With Adverse Maternal Outcomes in Women With Persistent Postpartum Hemorrhage. JAMA Netw Open.

[26] Wagner B, Meirowitz N, Shah J, Nanda D, Reggio L, Cohen P (2012). Comprehensive perinatal safety initiative to reduce adverse obstetric events. J Healthc Qual.

[27] Committee opinion no (2014). 590: preparing for clinical emergencies in obstetrics and gynecology. Obstet Gynecol.

[28] Kikuchi M, Itakura A, Miki A, Nishibayashi M, Ikebuchi K, Ishihara O (2013). Fibrinogen concentrate substitution therapy for obstetric hemorrhage complicated by coagulopathy. J Obstet Gynaecol Res.

[29] Matsunaga S, Takai Y, Seki H (2019). Fibrinogen for the management of critical obstetric hemorrhage. J Obstet Gynaecol Res.

[30] Seto S, Itakura A, Okagaki R, Suzuki M, Ishihara O (2017). An algorithm for the management of coagulopathy from postpartum hemorrhage, using fibrinogen concentrate as first-line therapy. Int J Obstet Anesth.

